# Assessment of natural variation in the first pore domain of the tomato HKT1;2 transporter and characterization of mutated versions of *SlHKT1;2* expressed in *Xenopus laevis* oocytes and via complementation of the salt sensitive *athkt1;1* mutant

**DOI:** 10.3389/fpls.2014.00600

**Published:** 2014-11-04

**Authors:** Pedro M. F. Almeida, Gert-Jan de Boer, Albertus H. de Boer

**Affiliations:** ^1^Department of Structural Biology, Faculty Earth and Life Sciences, Vrije Universiteit AmsterdamAmsterdam, Netherlands; ^2^R&D Department, Enza ZadenEnkhuizen, Netherlands

**Keywords:** natural variation, tomato, *HKT1* genes, mutation, pore domain

## Abstract

Single Nucleotide Polymorphisms (SNPs) within the coding sequence of HKT transporters are important for the functioning of these transporters in several plant species. To unravel the functioning of HKT transporters analysis of natural variation and multiple site-directed mutations studies are crucial. Also the *in vivo* functioning of HKT proteins, via complementation studies performed with *athkt1;1* plants, could provide essential information about these transporters. In this work, we analyzed the natural variation present in the first pore domain of the *HKT1;2* coding sequence of 93 different tomato accessions, which revealed that this region was conserved among all accessions analyzed. Analysis of mutations introduced in the first pore domain of the *SlHKT1;2* gene showed, when heterologous expressed in *Xenopus laevis* oocytes, that the replacement of S70 by a G allowed SlHKT2;1 to transport K^+^, but also caused a large reduction in both Na^+^ and K^+^ mediated currents. The study of the transport characteristics of SlHKT1;2 revealed that Na^+^-transport by the tomato SlHKT1;2 protein was inhibited by the presence of K^+^ at the outside of the membrane. *GUS* expression under the *AtHKT1;1* promoter gave blue staining in the vascular system of transgenic *Arabidopsis*. *athkt1;1* mutant plants transformed with *AtHKT1;1, SlHKT1;2, AtHKT1;1S68G*, and *SlHKT1;2S70G* indicated that both AtHKT1;1 and SlHKT1;2 were able to restore the accumulation of K^+^ in the shoot, although the low accumulation of Na^+^ as shown by WT plants was only partially restored. The inhibition of Na^+^ transport by K^+^, shown by the SlHKT1;2 transporter in oocytes (and not by AtHKT1;1), was not reflected in Na^+^ accumulation in the plants transformed with *SlHKT1;2*. Both AtHKT1;1-S68G and SlHKT1;2-S70G were not able to restore the phenotype of *athkt1;1* mutant plants.

## Introduction

Salinity stress negatively affects crop yield (Munns and Tester, 2008). In order to sustain the growing human population it is necessary to increase the salt tolerance of crop plants, as the human population is growing faster than the area of agricultural land (FAO, 2009). To tolerate salinity plants rely on three different mechanisms: osmotic tolerance, ionic tolerance and Na^+^ exclusion from the shoots (Munns and Tester, 2008). Na^+^ exclusion from the shoots is the most studied and best understood mechanism, therefore, it is a promising candidate for an approach of genetic modification to enhance plant salt tolerance (Plett et al., 2010).

HKT transporters are often studied with regard to Na^+^ exclusion from the shoots. HKT transporters belong to a superfamily of transporters including bacterial KtrBs transporters (Tholema et al., 1999) and yeast TRKs transporters (Rodriguez-Navarro, 2000). The *HKT* gene family is divided in two classes based on their gene structure and in the presence of either a glycine (G) or a serine (S) residue in the first pore domain of the transporter (Maser et al., 2002). Members of class 1 have an S at this position, whereas members of class 2, with the exception of OsHKT2;1, have a G at this position (Platten et al., 2006). HKT transporters are implicated in Na^+^ transport in wheat (Davenport et al., 2005; James et al., 2006; Byrt et al., 2007; Munns et al., 2012), rice (Ren et al., 2005; Horie et al., 2007; Jabnoune et al., 2009) and *Arabidopsis* (Uozumi et al., 2000; Berthomieu et al., 2003; Rus et al., 2004; Sunarpi et al., 2005; Moller et al., 2009). Class I HKT transporters are low affinity transporters with specificity for Na^+^ (Munns and Tester, 2008). Some of these members are located at the plasma membrane of root stele cells, in particular in the xylem parenchyma cells (XPC). They function in retrieving Na^+^ from the xylem sap, and prevent Na^+^ from reaching the shoots and damaging photosynthetic cells. The number of class I HKT members varies between mono- and dicotyledonous plants (Garciadeblas et al., 2003; Ren et al., 2005; Huang et al., 2006; Jabnoune et al., 2009). When first characterized, *athkt1;1* and wild type (WT) seedlings showed no difference in root and shoot growth after growing 6 days in a medium with (150 mM) or without NaCl (Rus et al., 2004). However, on the long term medium supplemented with 75 mM NaCl reduced the shoot growth and increased tip senescence of mature leaves of *athkt1;1* mutants (Rus et al., 2004). Due to the higher Na^+^ accumulation in the shoots, *athkt1;1* mutant plants display Na^+^ sensitivity, showing the role of HKT transporters in preventing Na^+^ from reaching the shoots (Rus et al., 2001, 2004; Berthomieu et al., 2003; Sunarpi et al., 2005).

The discovery of genetic polymorphisms in *HKT* genes (Diatloff et al., 1998; Rubio et al., 1999; Ren et al., 2005; Cotsaftis et al., 2012) underlying the adaptation to salinity stress brought new insights in the understanding of the functions of genes involved in the adaptation mechanisms. Information on genetic polymorphisms in *HKT* genes will provide tools for the development of crops more tolerant to salinity stress. Within the same genus, the diversity of phenotypes across environmental gradients of stress can indicate the suitability for selection, and the study of the genotypes responsible for those phenotypes might lead to the discovery of genetic polymorphisms responsible for these adaptive responses (Baxter et al., 2010). Analysis of *OsHKT1;5* in two rice cultivars differing in their salinity tolerance showed that both the cellular location and expression patterns of *OsHKT1;5* were identical. Nevertheless, differences in the coding region producing four amino acid substitutions were linked to the functional variation of these two alleles (Ren et al., 2005). Recently, it was concluded that the V395L substitution present in Nona Bokra could directly affect the Na^+^ transport rates and that it was responsible for the tolerant and sensitive behavior of Nona Bokra and Koshihikari, respectively (Cotsaftis et al., 2012).

Besides natural variation, studies of single (Diatloff et al., 1998; Maser et al., 2002) or multiple (Kato et al., 2007) site-directed mutations of HKT transporters (Diatloff et al., 1998; Maser et al., 2002; Kato et al., 2007) are also crucial to understand how these transporters function. The replacement of the S by a G in the first pore domain of AtHKT1;1 changed AtHKT1;1 from a Na^+^ uniporter to a Na^+^/K^+^ symporter (Maser et al., 2002).

Data on the role of HKT transporters is often generated from heterologous expression of HKT transporters in, mainly, *Xenopus laevis* oocytes (Schachtman and Schroeder, 1994; Fairbairn et al., 2000; Uozumi et al., 2000; Horie et al., 2001; Liu et al., 2001; Golldack et al., 2002; Berthomieu et al., 2003; Su et al., 2003; Ren et al., 2005; Jabnoune et al., 2009; Lan et al., 2010; Yao et al., 2010; Horie et al., 2011) and, to a lesser extent, in *Saccharomyces cerevisiae* cells (Horie et al., 2001, 2011; Golldack et al., 2002; Garciadeblas et al., 2003; Su et al., 2003; Takahashi et al., 2007; Ali et al., 2012). However, in a report by Haro et al. (2005) a very important question was addressed: are results obtained for HKT transporters when expressed in heterologous systems of physiological importance *in planta*? Or, are heterologous expression systems too artificial to have physiological meaning *in planta*? We performed several experiments to investigate if there is natural variation present in the first pore domain of HKT1;2 in tomato and how the replacement of the S residue by a G residue in the first pore domain in HKT1;2 of tomato affects the function of the transporter when expressed in *Xenopus laevis* oocytes. Moreover, we investigated if the results obtained with *Xenopus oocytes* could be replicated *in planta*.

In this study we analyzed the presence of natural variation in the first pore domain of HKT1;2 of 93 tomato accessions. We also replaced the S residue of the first pore domain of SlHKT1;2 with a G residue, and we analyzed the effect of this mutation via expression of these mutated genes in *Xenopus laevis* oocytes. We generated stable lines of *Arabidopsis thaliana athkt1;1* plants expressing each of these constructs, and we characterized their biomass production, shoot water-content and ion content after 2 weeks of salt treatment.

## Materials and methods

### Plant material and growth conditions

Genomic DNA used in this experiment was extracted from 93 different tomato accessions (Supplementary File Table 1). Tomato seeds were surface sterilized by soaking in 1% (V/V) commercial sodium hypochlorite solution for 15 min and rinsed three times with sterile water. After sterilization, seeds were sown in rock wool plugs soaked with half-strength Hoagland solution (one seed per rock wool plug). Plugs were covered with dry vermiculite to avoid dehydration. A randomized design consisting of three biological replicates and 93 different accessions was used. Each biological replica consisted of a pool of seven to 10 plants. On alternate days plants were irrigated with half-strength Hoagland solution. Plants were kept in a climate chamber under a 14/10 h photoperiod and a 20/18°C day/night temperature. Plants were harvested after 5 weeks. Seeds of homozygous *athkt1;1* mutant (Columbia-0) were obtained from the NASC stock center (N6531) and were sown along with WT *Arabidopsis thaliana* Col-0. Plants were grown on a mix of sand and peat (1:1) at 24°C in a 16 h light/8 h dark cycle in a greenhouse. Plants were watered every 2 days. Selected transgenic lines (See the section “Cloning of *HKT* genes and generation of *Arabidopsis* transgenic lines” for a full description of how these lines were generated) were grown under the same conditions. Four-week old transgenic lines (T2 lines) were treated with 100 mM NaCl every 2 days during 2 weeks before harvesting of shoot material.

### Genomic DNA extraction

For the extraction of genomic DNA approximately 25 mg of dried root material of each tomato accession was weighed and inserted in a 96 deep well plate. Samples were freeze dried for 1 week using a freeze dryer (Christ Alpha 1-4 LD plus, Germany). For the extraction of DNA a Nucleospin 96 Plant II kit (Macherey-Nagel, Germany) was used and the manufacturer's protocol was followed. The quality of the gDNA was checked on a 1.5% agarose gel. The concentration of the gDNA was calculated using the Quanti-iTTM PicoGREEN dsDNA assay kit (Invitrogen). gDNA was diluted in TE buffer pH 8.0 (10 mM Tris and 1 mM EDTA) and stored at 4°C.

### Primer and probe design and analysis of natural variation

Primers and probes (Supplementary File Table 2) were designed to have a Tm between 60 and 67°C with DNASIS MAX v3.0 software. Pairs of primers were designed to flank the SNP under study. The size of amplicons was designed to be smaller than 150 nucleotides. Unlabeled probes were blocked at the 3′ end to prevent extension in PCR reactions and designed to anneal over an SNP of interest. Reactions were performed in 384-well plates. 20 ng of gDNA per sample were used to study natural variation in tomato *HKT1;2* nucleotides of different cultivars. Per reaction, 3.21 μl of MilliQ, 0.05 μl of FW primer (10 μmol/μl) and 0.25 μl RV primer (10 μmol/μl), 0.04 μl of PAL (5 U/μl) (KAPA Biosystems, Boston, USA), 0.25 μl LC Green (Idaho Technology, Salt Lake City, USA), 0.20 μl dNTP (5 mM) and 1 μl PAL buffer (KAPA Biosystems, Boston, USA) supplemented with 12.5 mM MgCl_2_, were added. The amplification reaction started with a denaturation step of 95°C for 10 min and continued with 14 cycles of 95°C for 15 sec and 60°C for 4 min. Samples were cooled to room temperature and a first melting curve analysis was performed to assess the quality of the amplification. Samples were again cooled down to room temperature and 2 μl (10 pmol/μl) of probe was added. Samples were heated up to 96°C for 3 min and cooled down to room temperature before analyzing the melting curve of the probe. The mix of PCR products and probes was heated up to 96°C for 3 min and then cooled down to room temperature to allow hetero-duplex formation. Thereafter, the mix of PCR products and probes was re-heated to 95°C at 0.3°C/s. Data were acquired between 50 and 95°C. Data acquisition was made with a Light Scanner HR384 (Idaho Technology Inc. Salt Lake City, USA). High Resolution Melting (HRM) curve analysis was performed using the “Unlabeled Probes” module in the “genotyping” mode of the software. This mode involves negative filter, normalization and grouping. *SlHKT1;2* coding sequence isolated from *S. lycopersicum* Arbasson F1 was used as a reference. Amplicons that showed a melting curve different from the reference melting-curve were selected, amplified and sequenced. Sequencing of the amplicons was performed at Macrogen Europe Laboratories, Amsterdam, The Netherlands.

### Plasmid construction

Site-directed mutagenesis of *SlHKT1;2* was conducted using overlap extension PCR. All primers used to make mutated *HKT1* genes are listed in (Supplementary File Table 3). *pGEM-HESphI+SlHKT1;2* and *pGEM-HESphI+AtHKT1;1* (see Almeida et al., 2014 for a full description of how these constructs were obtained) were used as template and the corresponding mutated gene cloned into the BamHI and XbaI restriction sites and SphI and BamHI restriction sites of an empty *pGEM-HESphI* vector for tomato and *Arabidopsis HKT1*, respectively. The constructs obtained were called *pGEM-HESphI+AtHKT1;1-S68G* and *pGEM-HESphI+SlHKT1;2-S70G*. All PCR-derived DNA-fragments were confirmed by sequencing.

### Heterologous expression of *HKT1* genes in *Xenopus laevis* oocytes

Preparation of template DNA, *in vitro* transcription and capping of mRNA and Two-electrode voltage clamping of *Xenopus* oocytes was performed according to Almeida et al. (2014). All measurements were performed at least 3 times on oocytes isolated from two different batches.

### Cloning of *HKT* genes, generation of *Arabidopsis* transgenic lines and GUS staining

The *pGEM-HE* vector used in this work was modified to contain the *SphI* restriction site. This restriction site was introduced between the *BamHI* and *XbaI* restriction sites present in the vector. This was made by designing two complementary primers containing the *SphI* restriction site flanked by both *BamHI* and *XbaI* restriction sites, and introducing this piece of DNA between the *BamHI* and *XbaI* restriction sites of *pGEM-HE* vector. The *pGEM-HE* vector containing this new restriction site was selected and used in this work. To be able to distinguish the original *pGEM-HE* and *pGEM-HE* containing the *SphI* restriction site, we called our vector *pGEM-HESphI*.

*pGEM-HESphI+AtHKT1;1*, *pGEM-HESphI+AtHKT1;1-S68G*, *pGEM-HESphI+SlHKT1;2*, and *pGEM-HESphI+SlHKT1;2-S70G* were used as template in the amplification of the *HKT* genes flanked by the *attB* Gateway (Invitrogen) recombination sites. The *AtHKT1;1, AtHKT1;1-S68G, SlHKT1;2*, and *SlHKT1;2-S70G* genes were cloned into the *pDONR221 P5-P2* vector (Invitrogen) and were named *p5-2AtHKT1;1*, *p5-2AtHKT1;1-S68G*, *p5-2SlHKT1;2*, and *p5-2SlHKT1;2-S70G*, respectively. A 5 kb DNA fragment upstream of the *ATG* start codon of the *AtHKT1;1* gene containing the promoter region, the tandem repeat and the small RNA target region (Baek et al., 2011) was cloned into *pDONR221 P1-P5* and *pDONR221 P1–P2* (Invitrogen) and the resultant constructs were named *p1-5AtHKT1;1prom*, *p1-2AtHKT1;1prom*, respectively. Cloning of DNA fragments into *pDONR221* (Invitrogen) vectors was performed by BP reactions (Invitrogen). Cloning of either *AtHKT* or *SlHKT* genes into *pHGW* (Karimi et al., 2002) under the *AtHKT1;1* promoter was performed by LR reactions (Invitrogen). In this way, *pHGW+AtHKT1;1prom+AtHKT1;1*, *pHGW+AtHKT1;1prom+AtHKT1;1-S68G*, *pHGW+AtHKT1;1prom+SlHKT1;2*, and *pHGW+AtHKT1;1prom+SlHKT1;2-S70G* constructs were created. *p1-2AtHKT1;1prom* was incubated with *pKGWFS7* (Karimi et al., 2002) in an LR reaction (Invitrogen) to create the *pKGWFS7+AtHKT1;1prom* construct. All constructs were sequenced prior to the transformation of *Arabidopsis* plants. All primers used are listed (Supplementary File Table 4). All constructs were introduced into *Agrobacterium tumefaciens* strain *GV3101pMP90*, including the *pHGW* empty vector, and transformed into 3-week old *athkt1-1* mutant plants, except *pKGWFS7+AtHKT1;1prom* and the *pKGWFS7* empty vector, which were transformed into 3-week old *Arabidopsis* WT plants. Plant transformation was performed by the flower dipping method (Clough and Bent, 1998). *athkt1;1* mutant plants (N6531) (Rus et al., 2001) were transformed with *pHGW+AtHKT1;1prom+AtHKT1;1* or *pHGW+AtHKT1;1prom+AtHKT1;1-S68G* or *pHGW+AtHKT1;1prom+SlHKT1;2* or *pHGW+AtHKT1;1prom+SlHKT1;2-S70G. Arabidopsis* WT plants were transformed with *pKGWFS7+AtHKT1;1prom* or *pKGWFS7* empty vector. Four weeks after transformation, seeds were harvested and surface sterilized. Surface sterilization was performed by washing seeds during 1 min with a 80% ethanol solution with 0.1% Tween-20, followed by a 20 min washing step with 1% commercial bleach, and three washing steps with sterile MilliQ. MilliQ was then replaced by warm half-strength Murashige and Skoog (MS) medium supplemented with 1% sucrose, 0.8% agar and 10 mg/L hygromycin or 50 mg/L kanamycin. Seeds were placed in round plates containing solid MS medium with the same composition. Seedlings showing hygromycin or kanamycin resistance were selected and transferred to pots containing a mix of soil and peat (1:1). They were grown for 4 weeks under the growing conditions described above, after which the seeds were harvested. T2 seeds were tested for antibiotic resistance and used to either investigate their growth response under different NaCl concentrations or for GUS staining. Histochemical assays of GUS activity (biological replicates *n* = 3) were conducted using samples that were stained according to Jefferson et al. (1987). Seven days old plantlets (plantlets *n* = 7) were incubated at 37°C for 6 h in the staining solution containing 0.5 mM X-Gluc (5-bromo-4-chloro-3-indolyl-b-D-glucoronide).

### Analysis of transcripts levels

Tissue specific expression of all *HKT* genes was tested by extracting total RNA from roots and leaves of three-week old WT and transformed *Arabidopsis* lines. RNA from transformed plants was extracted and immediately frozen in liquid nitrogen. RNA extraction was performed using the Innuprep Plant RNA kit (Westburg, the Netherlands). First strand cDNA was synthesized using one microgram of total RNA, random hexamers and SuperScript II Reverse Transcriptase (Invitrogen Life Technologies). First strand cDNA was used as a template for quantitative real-time PCR (qRT-PCR). Samples were measured from each transformed plant using a sequence detector system (7300 Real Time PCR System from Applied Biosystems). Primers used to test the expression of *AtHKT1;1* and *AtHKT1;1-S68G* according to (Jha et al., 2010). Primers used to test the expression of *SlHKT1;2* and *SlHKT1;2-S70G* according to Asins et al. (2012). *ß-Actin* transcript levels were used as an internal standard. The mean normalized expression was calculated according to Livak and Schmittgen (2001).

### Statistical analyses of data

Two-way ANOVA was used to assess the effect of salt treatment on *HKT1* gene expression, fresh weight, Na^+^ and K^+^ accumulation. Normality and homogeneity assumptions were checked. Gene expression data were rank-transformed if these assumptions were violated. If ANOVA was significant, a *post hoc* test (Tukey's test) was used to evaluate differences among treatment and plant lines. All tests were performed using SPSS 17.0.

### Sequence data

The *SlHKT1;2* sequence used in this study has the EMBL/GenBank accession number HG530660 (Almeida et al., 2014).

## Results

### SLHKT1;2 S70 is conserved throughout *Solanum* accessions

**Figure 1:**
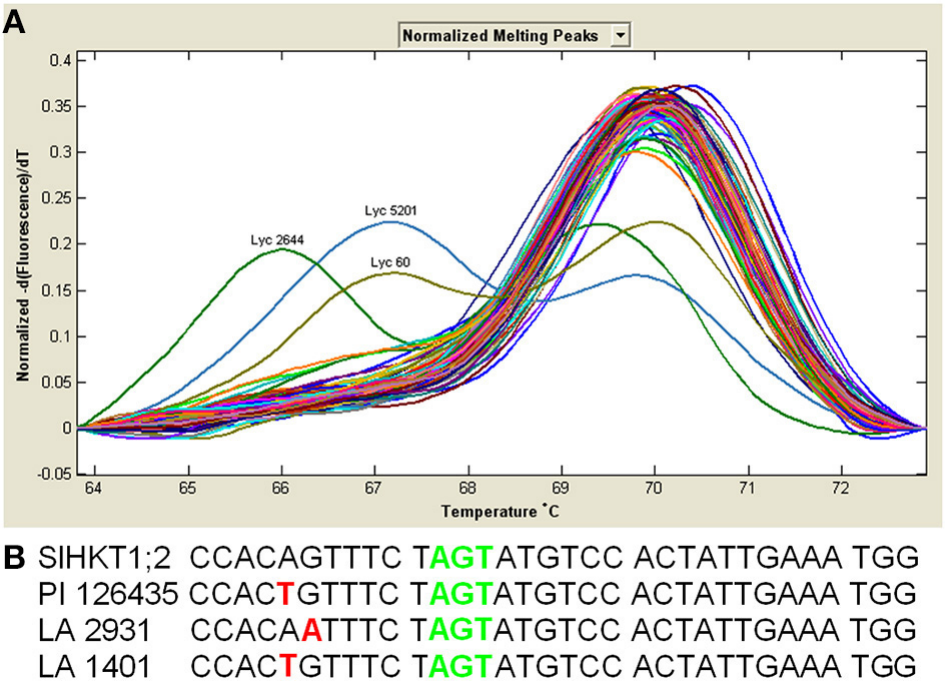
**Tomato accessions show SNPs close to the S at the filter position of the first pore domain, but not in the nucleotides coding the S residue. (A)** *Solanum HKT1;2* melting curve derivative plots from 93 tomato accessions showing the two different SNP's identified close to the 1st pore domain. **(B)** Nucleotide sequences of the *HKT1;2* gene of different *Solanum* accessions show that both SNPs (red letters) situate close to, but do not coincide with the S70 residue of the first pore domain (green letters). Black colored letters represent conserved nucleotides.

From all 93 tomato accessions tested only three (PI 126435, LA 2931 and LA 1401) showed different melting curves (Figure 1A). To identify whether the SNPs responsible for these different melting curves were located at our target region, we amplified and sequenced the region of the first pore domain of these three accessions (Figure 1B). Sequencing results revealed that each of these three accessions have a single SNP (PI 126435 and LA 1401 have the same SNP and LA 2931 has a different SNP), although none of them in the position of interest. The SNP in the accession LA 2931 resulted in an amino acid change from valine (V) to isoleucine (I); however, both amino acids are hydrophobic. In the case of accessions PI 126435 and LA 1401 the SNP did not result in any amino acid change, as both ACA and ACT code for a threonine (T) residue.

### Site-directed mutagenesis of S70 to G of SLHKT1;2

**Figure 2:**
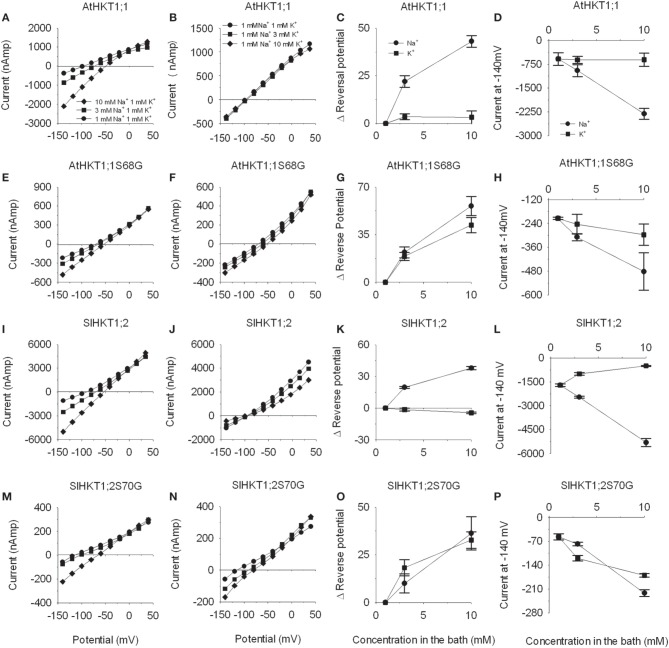
**Transport activity of *AtHKT1;1*, *AtHKT1;1-S68G*, *SlHKT1;2* and *SlHKT1;2-S70G* constructs expressed in *Xenopus laevis* oocytes.** Oocytes were injected with *AtHKT1;1* and *SlHKT1;2* and the indicated mutated transporters. **(A,E,I,M)** Currents recorded at three Na^+^ concentrations (1, 3, and 10 mM) with 1 mM K^+^ as background; **(B,F,J,N)** Currents recorded at three K^+^ concentrations (1, 3, and 10 mM) with 1 mM Na^+^ as background; **(C,G,K,O)** Reversal potential shifts as a function of ion concentration. Only transporters where the S of the 1st pore domain was mutated to a G were permeable to K^+^ as indicated by the large positive shifts in the reversal potential with increasing concentrations of K^+^ in the bath; **(D,H,L,P)** Absolute currents as a function of ion concentration. Transporters where the S of the 1st pore domain was mutated to a G showed an increase in current with increasing K^+^ concentration in the bath. Data are means ± *SE* (*n* = 3, experiments done with at least two different oocyte batches).

To test the hypothesis that S70 of the tomato SlHKT1;2 protein is crucial for the Na^+^ selectivity we replaced S70 by a G (SlHKT1;2-S70G). cRNA of *SlHKT1;2-S70G* was injected in *Xenopus laevis* oocytes. After 2 days of incubation, currents produced in the presence of Na^+^ and K^+^ ions were recorded in oocytes expressing both WT and mutated HKT1 transporters from *Arabidopsis thaliana* and *Solanum lycopersicum* (*n* = 3) (Figure 2). *AtHKT1;1-S68G* expressing oocytes (Maser et al., 2002) were used as a positive control of SlHKT1;2-S70G. Currents produced by oocytes expressing either *AtHKT1;1* (Figure 2A) or *SlHKT1;2* (Figure 2I) increased when the oocytes were bathed in higher Na^+^ concentrations (as seen by a more negative current). Increasing external K^+^ concentration did not result in any change in the current levels produced by *AtHKT1;1* expressing oocytes (Figure 2B). In contrast, *SlHKT1;2* mediated inward and outward currents were sensitive to external K^+^ concentration as both currents decreased with increasing bath K^+^ concentration (Figure 2J). Increased concentrations of K^+^ result in an inhibition on the transport of Na^+^ by SlHKT1;2 but not by AtHKT1;1. When oocytes expressing either *AtHKT1;1-S68G* or *SlHKT1;2-S70G* were bathed with either increasing Na^+^ concentration (Figures 2E,M) or K^+^ concentration (Figures 2F,N) currents increased for both cations tested. For both AtHKT1;1- and SlHKT1;2-mediated currents a higher Na^+^ concentration but not a higher K^+^ concentration resulted in positive shifts in the reversal potential (Figures 2C,K), which is indicative of Na^+^ permeation. Reversal potentials obtained with oocytes expressing either *AtHKT1;1-S68G* (Figure 2G) or *SlHKT1;2-S70G* (Figure 2O) showed positive shifts when both Na^+^ concentration or K^+^ concentration increased, indicating that the presence of a G residue at the filter position of the first pore domain allows the transport of both Na^+^ and K^+^ ions. Figures 2D,H,L,P show the currents recorded at −140 mV for AtHKT1;1, AtHKT1;1-S68G, SlHKT1;2 and SlHKT1;2-S70G, respectively. These results show that the Na^+^-mediated current of SlHKT1;2 is reduced by increased concentrations of K^+^ in the bath. The presence of K^+^ ions affects the transport of Na^+^ by SlHKT1;2. This effect is not observed with AtHKT1;1.

### Selection and molecular analysis of T2 plants and GUS expression in *A. thaliana* under the *A. thaliana* HKT1;1

**Figure 3:**
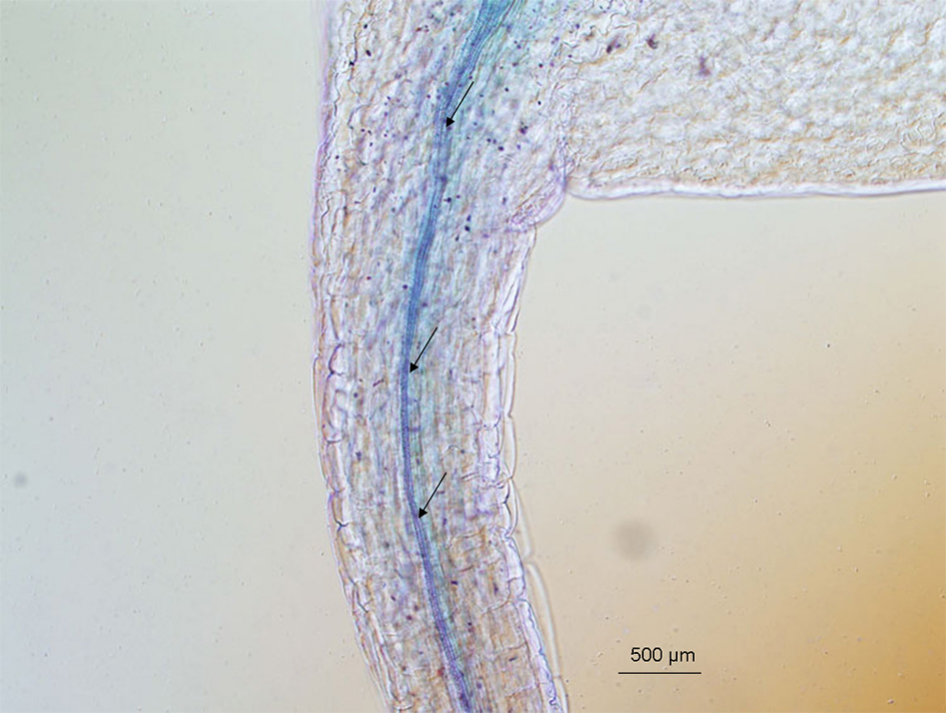
**Detection of GUS activity in the vascular system of transgenic *Arabidopsis thaliana* plants.** *Arabidopsis* plants expressed the *GUS* gene under the control of the *AtHKT1;1* promoter were grown. Strong blue GUS staining was detected in the vicinity of the xylem and phloem in leaves. Arrows point to the xylem vessels. Biological replicates, *n* = 3; plantlets, *n* = 7.

*HKT1* gene expression was analyzed in T2 *Arabidopsis* plantlets carrying the *SlHKT1;2* or *AtHKT1;1, SlHKT1;2-S70G*, or *AtHKT1;1-S68G* genes (data not shown). All genes were expressed under the *AtHKT1;1* endogenous promoter. All lines tested showed *HKT1* expression levels higher than the *athkt1;1* (N6531) mutant plants. Expression of *AtHKT1;1* was not detected in *athkt1;1* mutant plants. The 5 kb *AtHKT1;1* promoter fragment was able to drive the expression of *GUS* in the vascular tissues of transformed *Arabidopsis* WT plants (Figure 3). GUS activity driven by *AtHKT1;1* promoter was only observed in the vascular tissues of leaves of transgenic *Arabidopsis* plants (Figure 3).

### Analysis of FW, Na^+^, and K^+^ accumulation of transgenic *Arabidopsis* lines

**Figure 4:**
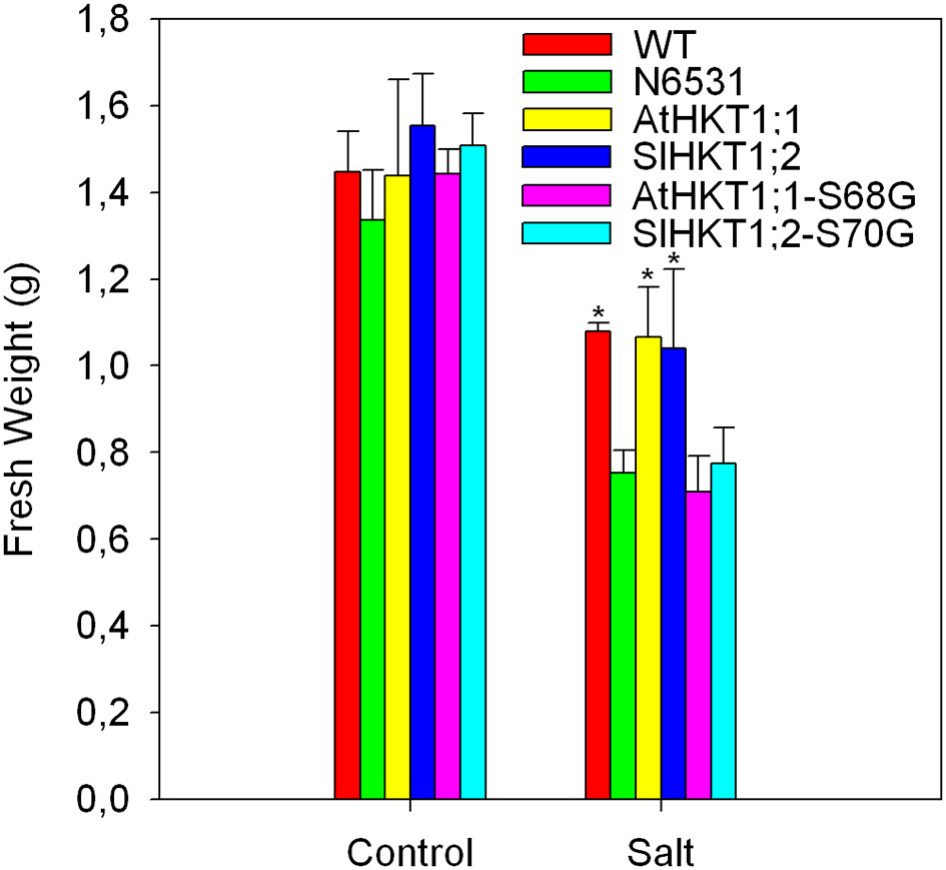
**Presence of 100 mM NaCl in the irrigation water during 2 weeks significantly reduced the fresh weight of transformed, WT and *athkt1;1* (N6531) *Arabidopsis* plants in comparison to control plants irrigated with water not supplemented with NaCl.** Different inhibitions on the fresh weight production are observed amongst different transformed plant lines. *Arabidopsis* WT and *athkt1;1* plants were used as positive and negative controls, respectively. Treatment: *p* < 0.05; plant lines: *p* < 0.05; treatment^*^plant lines (n.s.); Two-Way ANOVA. Values indicate the means ± *SE* of three to seven biological replicates.

**Figure 5:**
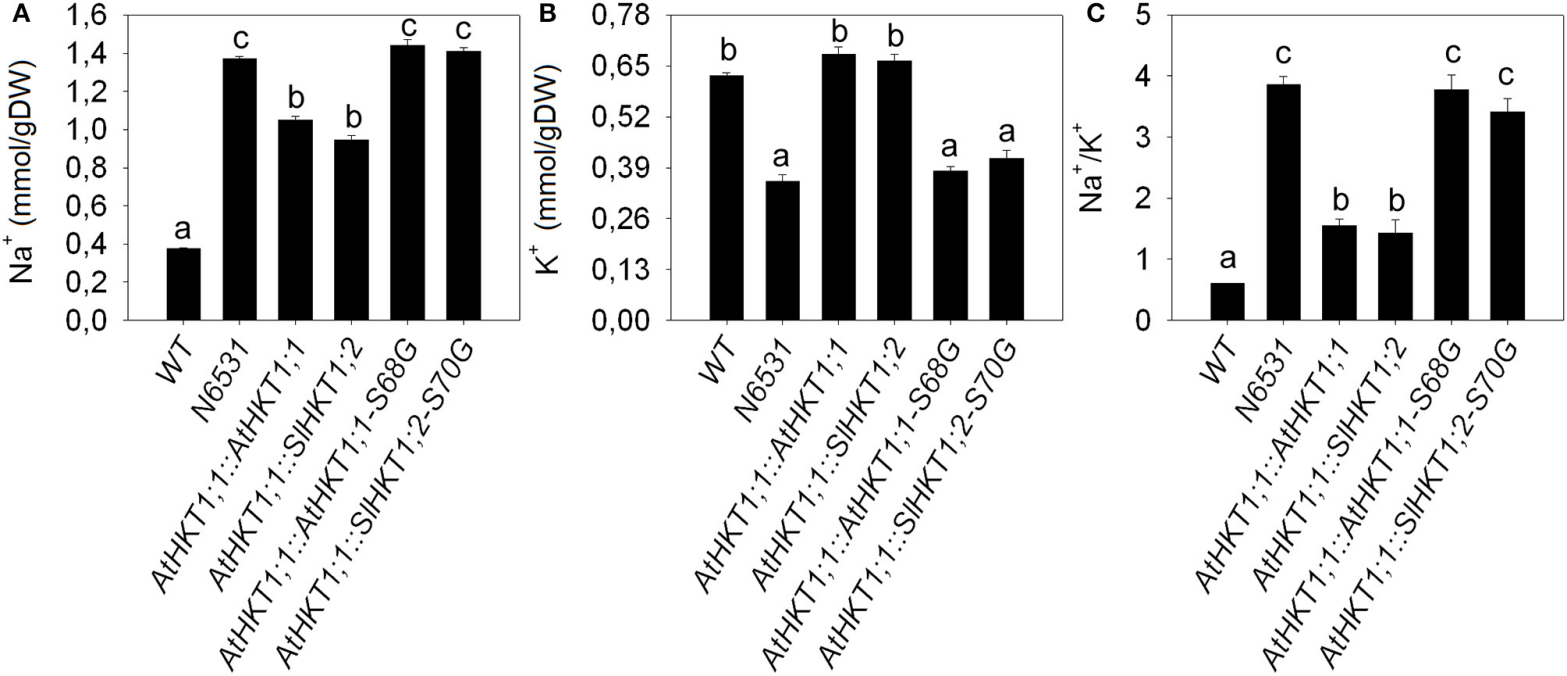
**Differences in ion accumulation between all plants analyzed.** Na^+^ **(A)** and K^+^ **(B)** accumulation and Na^+^/K^+^ **(C)** ratio in the shoot of WT, *athkt1;1* mutant (N6531) and transgenic lines expressing different *HKT1* genes. Na^+^/K^+^ ratio was normalized for WT. The effect of the mutation in the *AtHKT1;1* gene is very clear, showing a strong increase in shoot Na^+^ accompanied by a decrease in K^+^, which resulted in a very strong increase in the Na^+^/K^+^ ratio. Both lines expressing *AtHKT1;1*::*AtHKT1;1* or *AtHKT1;1*::*SlHKT1;2* were able to reduce the accumulation of Na^+^ and increase the accumulation of K^+^ in comparison to *athkt1;1*. Lines expressing *AtHKT1;1*::*AtHKT1;1-S68G* or *AtHKT1;1*::*SlHKT1;2-S70G* were not able to ameliorate the phenotype of the *athkt1;1* plants. Different letters above bars indicate statistically significant differences. Values indicate the means ± *SE* of three to seven biological replicates.

There was a marked difference in K^+^-sensitivity of Na^+^-transport mediated by the AtHKT1;1 and SlHKT1;2 proteins, as analyzed in *Xenopus laevis* oocytes. To see whether this difference was reflected in Na^+^/K^+^ homeostasis *in planta*, we expressed the *AtHKT1;1* and *SlHKT1;2* genes driven by a 5 kb long *AtHKT1;1* promoter in the *athkt1;1* mutant plants and studied how they complemented the mutant phenotype. Since the analysis of *SlHKT1;2-S70G* and *AtHKT1;1-S68G* in *Xenopus* oocytes showed interesting effects on transport activity and ion selectivity we transformed *athkt1;1* plants with *SlHKT1;2*-S70G and *AtHKT1-S68G*, and tested the functioning of these mutated genes *in planta* during salinity stress. Figure 4 shows the effect of salinity treatment on the leaf fresh weight of WT plants, *athkt1;1* mutants and transformed plants. *athkt1;1-*mutant plants were more sensitive to salt than the WT plants. Both the *AtHKT1;1* and the *SlHKT1;2* gene were able to complement the *athkt1;1* mutant growth-phenotype on salt (Figure 4). Plants transformed with *SlHKT1;2*-S70G or *AtHKT1-S68G* were just as sensitive to salt as the *athkt1;1-*mutant plants. The analysis of the relative water content of the leaves did not show statistically significant differences between control and salt treated plants (data not shown) nor within transformed lines. The effect of the *athkt1;1* mutation on Na^+^ and K^+^ homeostasis was pronounced (Figure 5). The *athkt1;1* plants accumulated almost four-fold more Na^+^ and two-fold less K^+^, resulting in an eight-fold higher Na^+^/K^+^-ratio in the shoot of the mutant plants as compared to the WT plants. The transgenic lines that complemented the *athkt1;1* growth-phenotype (*AtHKT1;1* and *SlHKT1;2*) showed effects on ion accumulation: the *athkt1;1* K^+^-phenotype during salt stress (i.e., strong reduction in K^+^ accumulation) was completely restored, but Na^+^ accumulation in the leaves of these transgenic lines was still significantly higher than that of the WT plants.

## Discussion

Several studies have shown that naturally occurring SNPs in genes involved in Na^+^ and K^+^ homeostasis can have a dramatic effect on the salinity tolerance of several plant species. This is the case for HKT transporters (Rubio et al., 1995; Ren et al., 2005; Baxter et al., 2010; Ali et al., 2012; Cotsaftis et al., 2012). Due to the effects of SNPs on the transport properties of HKT proteins, and consequently the effect of these SNPs on the salinity tolerance of the plants, we decided to study the presence/absence of SNPs in the sequence of the first pore domain of the *HKT1;2* gene of several tomato accessions (Figure 1). The first pore domain in all accessions studied showed an S, which is naturally occurring in AtHKT1;1, implying that if during evolution the amino acid residue that we studied would have changed, these changes were not in favor of salinity tolerance of the tomato plants and, therefore, did not persist (Diatloff et al., 1998).

**Figure 6:**
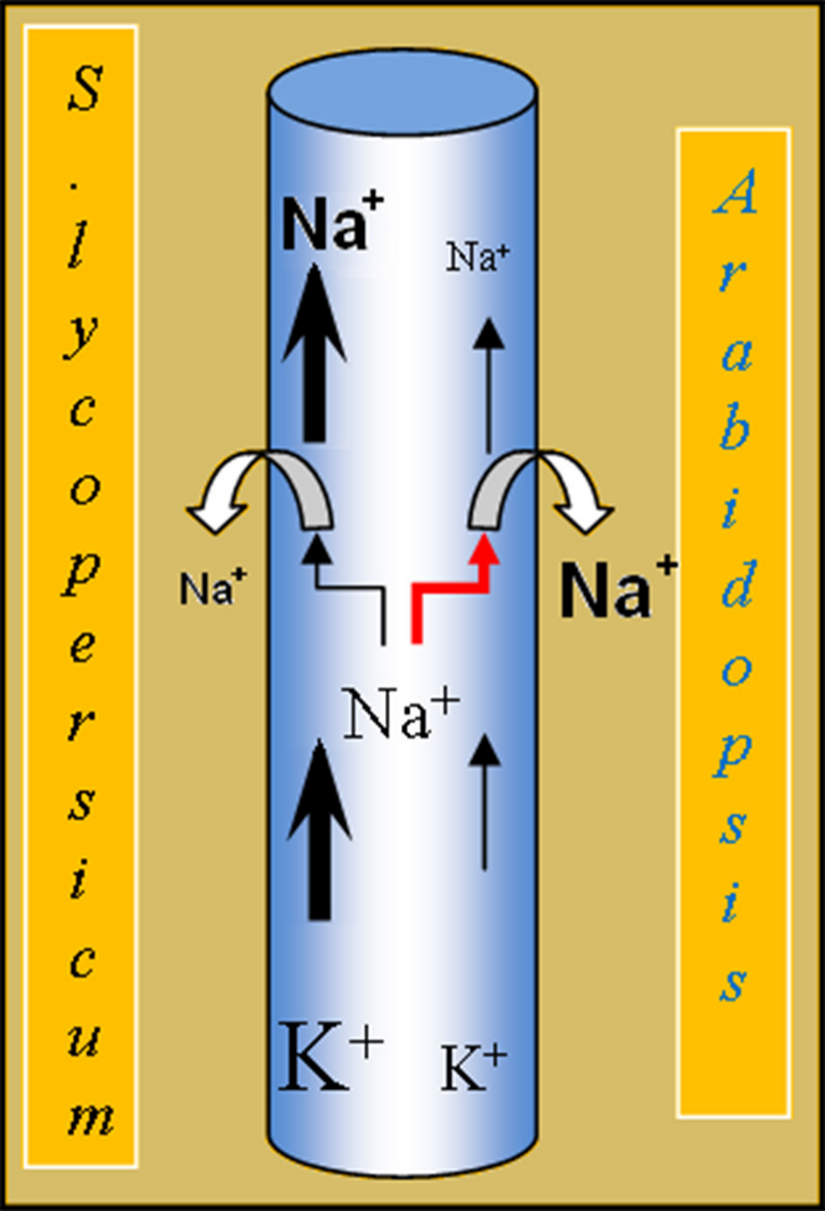
**Model depicting the difference in K^+^-sensitivity of SlHKT1;2 from *S. esculentum* and AtHKT1;1 from *Arabidopsis*.** When the K^+^-concentration in the xylem sap is high, Na^+^-uptake by the SlHKT1;2 transporter is reduced. As a result the amount of Na^+^ in the xylem stream reaching the shoot of tomato is, at least partially, controlled by SlHKT1;2, which in turn depends on the concentration of K^+^ present in the xylem sap. In *Arabidopsis*, Na^+^-uptake in the XPCs by the AtHKT1;1 transporter is not affected by high K^+^.

The analysis of the properties of the heterologous expressed *SlHKT1;2* showed that the transport characteristics were in accordance with the presence of an S in the first pore domain of the transporter (Figure 2). This is reflected in the SlHKT1;2 transport characteristics as measured in heterologous expression, where it was shown that tomato HKT1;2 transports Na^+^ but not K^+^ (Almeida et al., 2014). However, a striking difference between the transport properties of AtHKT1;1 and SlHKT1;2 expressed in oocytes was observed when currents were measured at constant Na^+^ in the bath (1 mM) and increasing K^+^ (1, 3, and 10 mM). Whereas the AtHKT1;1 mediated current was virtually insensitive to higher K^+^, the SlHKT1;2 mediated current decreased by 60% at 10 mM K^+^ (Figure 2). A similar inhibitory action of K^+^ on HKT-mediated currents was reported for OsHKT2;1 (Jabnoune et al., 2009) and for TmHKT1;5-A (Munns et al., 2012). It was proposed that this inhibition is caused by the association of K^+^ to the Na^+^ binding region within the pore region of HKT transporters (Rubio et al., 1995; Gassmann et al., 1996). This inhibition has not been observed with AtHKT1;1 (Uozumi et al., 2000) nor OsHKT1;5 (Ren et al., 2005) in *Xenopus* oocytes, which indicates that the K^+^-sensitivity of these transporters is different from OsHKT2;1, TmHKT1;5-A and tomato SlHKT1;2. Physiologically, this K^+^-induced reduction of Na^+^-influx might mean that the tomato plants maintain a certain K^+^/Na^+^-homeostasis in the transpiration sap. High concentrations of K^+^ in the xylem sap might imply a reduced Na^+^-uptake into the XPC, as the Na^+^/K^+^ ratio is in favor of Na^+^. On the other hand, low concentrations of K^+^ and high concentrations of Na^+^ in the xylem sap imply an induced Na^+^-uptake into the XPC, as the Na^+^/K^+^ ratio is in favor of K^+^ in the xylem sap. In this model, the Na^+^/K^+^ ratio is more important than the absolute concentration of Na^+^ in the xylem sap in determining the Na^+^ uptake into the XPC by HKT1 (Figure 6). Although the S → G mutation in the first pore domain of both the AtHKT1;1 and SlHKT1;2 protein had an effect on the ion selectivity of both transporters, as deduced from the shift in reversal potential at increasing external K^+^ (Figure 2), a major difference observed was the reduction in total current transported by SlHKT1;2-S70G and AtHKT1-S68G, which was 95 and 78% respectively of that transported by the WT proteins at 10 mM Na^+^ and 1 mM K^+^ in the bath (Figure 2). The reason for this difference is unclear as the results we obtained do not match the results of the Na^+^ currents produced by AtHKT1;1-S68G reported by Maser et al. (2002). Their results did not show this reduction in total currents. Interestingly, when Maser et al. (2002) mutated AtHKT1;1 in M69L, a reduction in outward currents with increasing K^+^ concentrations in the bath was observed, whereas in TaHKT1;2 the reverse mutation, L92M, abolished this inhibitory effect of K^+^ on outward currents (Maser et al., 2002). In their work, the leucine (L) adjacent to the G of the pore domain seems to confer K^+^ sensitivity to outward currents in contrast with the methionine (M) at that same position, which abolished the effect of K^+^ on Na^+^ currents (Maser et al., 2002). Since in our study SlHKT1;2 has a M adjacent to the G of the pore domain, no sensitivity of outward currents was expected due to increasing K^+^ concentrations in the bath. Nevertheless, in our study we observed outward currents, mediated by SlHKT1;2, sensitive to external K^+^. In a future study it would be interesting to mutate SlHKT1;2-M71L, to check whether the presence of a L adjacent to the S of the first pore domain of SlHKT1;2 enhances or decreases this inhibitory effect of external K^+^ on the outward Na^+^-currents mediated by SlHKT1;2.

### *ATHKT1;1* and *SLHKT1;2* expressing lines

All plant lines studied showed a reduction in fresh weight caused by the salt treatment, and as expected the salt-induced growth reduction was stronger in the *athkt1;1*-mutant plants than in WT plants. With respect to growth on salt, *AtHKT1;1* and *SlHKT1;2* expression complemented the mutant, since the fresh weight of these lines was comparable to that of WT plants treated with salt and significantly higher than the growth of *athkt1;1* plants (Figure 4). Although expression of *AtHKT1;1* and *SlHKT1;2* completely restored the concentration of K^+^ in the shoot to the same level of the WT plants, Na^+^ levels were still higher in *AtHKT1;1* and *SlHKT1;2*-expressing lines in comparison to WT plants. Both *AtHKT1;1* and *SlHKT1;2* expressing lines accumulated significantly less Na^+^ than the other transgenic lines, but significantly more Na^+^ than the WT plants. This indicates that in these two transgenic lines both *HKT1* genes do not retrieve the same amount of Na^+^ from the xylem as WT plants. This difference in Na^+^ accumulation in lines expressing *AtHKT1;1* and *SlHKT1;2* in comparison to WT plants is surprising. In this study we used the native *AtHKT1;1* promoter (Baek et al., 2011) to drive the expression of all genes studied, avoiding non-native promoters as these are frequently referred to as the cause of unexpected results (Cominelli and Tonelli, 2010). The expression of *GUS* driven by the native *AtHKT1;1* promoter (Figure 3) showed that the expression patterns were similar to previous results (Sunarpi et al., 2005) and also the level of *HKT*-expression in the different transgenic lines was comparable to that in WT plants.

When expressed in *Xenopus laevis* oocytes *AtHKT1;1* and *SlHKT1;2* differed in two ways: first, SlHKT1;2 but not AtHKT1;1 showed an inhibition of Na^+^ transport by K^+^, and second, the total Na^+^-mediated current (i.e., the turn-over) measured in *SlHKT1;2* expressing oocytes was considerably higher than the total Na^+^-mediated current in *AtHKT1;1* expressing oocytes (Figure 2). The latter conclusion needs further testing, since it is based on the assumption that injection of the same amount of cRNA results in the same amount of protein expressed in the plasma membrane. However, it is clear that in these two transgenic lines both HKT1 transporters are involved in the retrieval of Na^+^ ions from the xylem, as previously demonstrated for HKT1 transporters from several species (Liu et al., 2001; Laurie et al., 2002; Berthomieu et al., 2003; Garciadeblas et al., 2003; Ren et al., 2005; Horie et al., 2007; Jabnoune et al., 2009; Moller et al., 2009; Baxter et al., 2010; Plett et al., 2010; Baek et al., 2011; Mian et al., 2011; Ali et al., 2012; Munns et al., 2012).

As shown in Figure 5, mutating the *athkt1;1* gene not only results in an increase in shoot Na^+^ concentration, but also in a strong reduction in shoot K^+^ levels. This effect on K^+^-accumulation has been reported before (Rus et al., 2004, 2005), but a good explanation for this effect is missing thus far. Moller et al. (2009) reported that the increase in K^+^ concentration in the shoots from plants over-expressing *AtHKT1;1*, was a pleiotropic effect and a consequence of the reduced Na^+^ shoot content. Another explanation is that the uptake of Na^+^ from the xylem into the XPCs via HKT1 results in the depolarization of the membrane potential of XPCs and activation of the depolarization activated K^+^-efflux channel SKOR, resulting in more K^+^ release into the xylem (Sunarpi et al., 2005). A third explanation might be that AtHKT1;1 functionally interacts with a K^+^-efflux transporter in the plasma membrane of XPCs. Support for this hypothesis is found in a recently published large-scale membrane interaction screen based on a yeast mating split-ubiquitin system (mbSUS) (Membrane-based Interactome Network Database, MIND: http://cas-biodb.cas.unt.edu/project/mind/index.php). In this screen, AtHKT1;1 was reported to interact with KEA3, a putative K^+^-efflux antiporter and member of the Proton Antiporter-2 (CPA2) family. However, this needs to be confirmed *in planta* in order to provide an explanation for the K^+^-phenotype of the *athkt1;1* mutant. The absence of the AtHKT1;1 protein in the plasma membrane of XPCs of *athkt1;1* plants may have a negative effect on the KEA3 antiporter, resulting in reduced root to shoot K^+^-transport. These hypotheses are certainly worth testing in view of the importance of Na^+^/K^+^-homeostasis during salinity stress.

In conclusion, the analysis of the presence of SNPs in the first pore domain of the *HKT1;2* gene in 93 tomato accessions showed that this amino acid is conserved in all these accessions. The replacement of S70 by a G in SlHKT1;2 proved to be sufficient to alter the transport specificity of this transporter, as analyzed by heterologous expression in *Xenopus laevis* oocytes. Moreover, when expressed under the *AtHKT1;1* native promoter, both *AtHKT1;1* and *SlHKT1;2* partially complemented the Na^+^ accumulation phenotype and fully complemented the K^+^ accumulation phenotype of *athkt1;1* mutant plants. *AtHKT1;1-S68G* and *SlHKT1;2-S70G* were unable to complement either the Na^+^ or the K^+^ phenotype of *athkt1;1* mutant plants. The transport activity of mutated HKT proteins, measured in *Xenopus* oocytes, was not reflected in altered Na^+^/K^+^-homeostasis of the *athkt1;1* mutants grown under soil conditions.

### Conflict of interest statement

The authors declare that the research was conducted in the absence of any commercial or financial relationships that could be construed as a potential conflict of interest.
