# Supplementary material for: Assessment of natural variation in the first pore domain of the tomato HKT1;2 transporter and characterization of mutated versions of SlHKT1;2 expressed in Xenopus laevis oocytes and via complementation of the salt sensitive athkt1;1 mutant
Source: Front Plant Sci. 2014 Nov 4;5:600. doi: 10.3389/fpls.2014.00600 (PMC4219482; doi:10.3389/fpls.2014.00600)
Supplement: Supplementary file 1 [file Table1.DOCX]

**Supplementary File Table 1: Names of all accessions tested in experiment 1.**

| Accession | Source | Species/Name | Acc. number |
| --- | --- | --- | --- |
| Arbasson F1 | ENZA Zaden | *S. lycopersicum* | 1 |
| Elpida F1 | ENZA Zaden | *S. lycopersicum* | 2 |
| Estrella F1 | ENZA Zaden | *S. lycopersicum* | 3 |
| Hybrid | ENZA Zaden | *S. lycopersicum* | 4 |
| Newton F1 | ENZA Zaden | *S. lycopersicum* | 5 |
| G 1560 | IVT | *S. habrochaites* | 6 |
| LA 2194 | TGRC | *S. neorickii* | 7 |
| LA 2774 | TGRC | *S. chilense* | 8 |
| LA 0247 | TGRC | *S. neorickii* | 9 |
| OT.2209 | IVT | *S. pimpinellifolium* | 10 |
| PI 128653 | Vavilov | *S. peruvianum* | 11 |
| PI 247087 | INRA | *S. habrochaites* | 12 |
| PI 126440 | USDA | *S. corneliomuelleri* | 13 |
| LA 1401 | TGRC | *S. galapagense* | 14 |
| LA 1028 | TGRC | *S. chmielewskii* | 15 |
| LA 1316 | TGRC | *S. chmielewskii* | 16 |
| GI 568 | IVT | *S. corneliomuelleri* | 17 |
| LA 1938 | TGRC | *S. chilense* | 18 |
| LA 1959 | TGRC | *S. chilense* | 19 |
| PI 128659 | USDA | *S. peruvianum* | 20 |
| LA 1508 | TGRC | *S. galapagense* | 21 |
| LA 1412 | TGRC | *S. cheesmaniae* | 22 |
| LA 1248 | TGRC | *S. pimpinellifolium* | 23 |
| LA 1259 | TGRC | *S. pimpinellifolium* | 24 |
| LA 1243 | TGRC | *S. pimpinellifolium* | 25 |
| LA 1306 | TGRC | *S. chmielewskii* | 26 |
| LA 2917 | TGRC | *S. neorickii* | 27 |
| LA 1319 | TGRC | *S. neorickii* | 28 |
| LA 1325 | TGRC | *S. chmielewskii* | 29 |
| LA 2639B | TGRC | *S. chmielewskii* | 30 |
| LA 2678 | TGRC | *S. chmielewskii* | 31 |
| LA 2681 | TGRC | *S. chmielewskii* | 32 |
| LA 2695 | TGRC | *S. chmielewskii* | 33 |
| LA 0531 | TGRC | *S. cheesmaniae* | 34 |
| LA 0532 | TGRC | *S. galapagense* | 35 |
| LA 0317 | TGRC | *S. galapagense* | 36 |
| LA 1961 | TGRC | *S. chilense* | 37 |
| LA 2747 | TGRC | *S. chilense* | 38 |
| LA 3320 | TGRC | *S. lycopersicum* | 39 |
| LA 2662 | TGRC | *S. lycopersicum* | 40 |
| LA 3120 | TGRC | *S. lycopersicum* | 41 |
| LA 1363 | AVRDC | *S. habrochaites* | 42 |
| PI 126443 | USDA | *S. corneliomuelleri* | 43 |
| PI 407543 | USDA | *S. pimpinellifolium* | 44 |
| PI 407546 | USDA | *S. pimpinellifolium* | 45 |
| PI 134417 | USDA | *S. habrochaites glabratum* | 46 |
| CGN19145 | IVT | *S. minutum* | 47 |
| LA 2744 | TGRC | *S. peruvianum* | 48 |
| LA 1033 | TGRC | *S. habrochaites* | 49 |
| LA 1223 | TGRC | *S. pimpinellifolium* | 50 |
| PI 126935 | USDA | *S. peruvianum* | 51 |
| LA 2981A | TGRC | *S. chilense* | 52 |
| LA 1245 | TGRC | *S. pimpinellifolium* | 53 |
| Abigail F1 | Hazera Genetics | *S. lycopersicum* | 54 |
| Daniela | Hazera Genetics | *S. lycopersicum* | 55 |
| EZ-8 | ENZA Zaden | *S. lycopersicum* | 56 |
| LA 1302 | TGRC | *S. pennellii puberulum* | 57 |
| LA 1340 | TGRC | *S. pennellii* | 58 |
| LA 1522 | TGRC | *S. pennellii* | 59 |
| LA 1624 | TGRC | *S. habrochaites glabratum* | 60 |
| LA 1965 | TGRC | *S. chilense* | 61 |
| LA 1971 | TGRC | *S. chilense* | 62 |
| LA 2167 | TGRC | *S. habrochaites* | 63 |
| LA 2548 | TGRC | *S. peruvianum* | 64 |
| LA 2560 | TGRC | *S. pennellii* | 65 |
| LA 2748 | TGRC | *S. chilense* | 66 |
| LA 2750 | TGRC | *S. chilense* | 67 |
| LA 2860 | TGRC | *S. habrochaites glabratum* | 68 |
| LA 2879 | TGRC | *S. chilense* | 69 |
| LA 2931 | TGRC | *S. chilense* | 70 |
| 964750062 | Hortus Botanicus Nijmegen | *S. neorickii* | 71 |
| CGN15879 | CGN | *S. habrochaites glabratum* | 72 |
| PI 126449 | USDA | *S. habrochaites glabratum* | 73 |
| LA 1910 | TGRC | *S. peruvianum* | 74 |
| LA 2773 | TGRC | *S. chilense* | 75 |
| PI 126435 | USDA | *S. peruvianum* | 76 |
| LA 0462 | TGRC | *S. peruvianum* | 77 |
| LA 3218 | TGRC | *S. peruvianum* | 78 |
| LA 1930 | TGRC | *S.chilense* | 79 |
| LA 2711 | TGRC | *S.lycopersicum* | 80 |
| LA 1278 | TGRC | *S.peruvianum* | 81 |
| LA 1310 | TGRC | *S.lycopersicum cerasiforme* | 82 |
| LA 1393 | TGRC | *S.habrochaites* | 83 |
| LA 1579 | TGRC | *S.pimpinellifolium* | 84 |
| LA 2874 | TGRC | *S.lycopersicum cerasiforme* | 85 |
| LA 2880 | TGRC | *S.chilense* | 86 |
| EZ-1 | ENZA Zaden | *S.lycopersicum* | 87 |
| EZ-2 | ENZA Zaden | *S.lycopersicum* | 88 |
| EZ-3 | ENZA Zaden | *S.lycopersicum* | 89 |
| EZ-4 | ENZA Zaden | *S.lycopersicum* | 90 |
| EZ-5 | ENZA Zaden | *S.lycopersicum* | 91 |
| EZ-6 | ENZA Zaden | *S.lycopersicum* | 92 |
| EZ-7 | ENZA Zaden | *S.lycopersicum* | 93 |
