# Supplementary material for: Assessment of natural variation in the first pore domain of the tomato HKT1;2 transporter and characterization of mutated versions of SlHKT1;2 expressed in Xenopus laevis oocytes and via complementation of the salt sensitive athkt1;1 mutant
Source: Front Plant Sci. 2014 Nov 4;5:600. doi: 10.3389/fpls.2014.00600 (PMC4219482; doi:10.3389/fpls.2014.00600)
Supplement: Supplementary file 2 [file Table2.DOCX]

**Supplementary File Table 2:** List of primers and probes used to study the presence or absence of SNPs in *Solanum sp.* gDNA sequence regions where amino acids with important roles in the functioning of the transporter were identified in other plant species.

| **Nucleotide tested** | **Primer name** | **Primer sequence (5’ to 3’)** |
| --- | --- | --- |
| SlHKT1;2-S70 | SlHKT1;2 Fw | CCTAGAACCCTACCGTC |
|  | SlHKT1;2 Rv | GAACATTTGAGAAAACTTCC |
|  | SlHKT1;2 P | CCACAGTTTCTAGTATGTCCACTATTGAAATGC |
| AtHKT1;1-S68 | AtHKT1;1 Fw | CAAGAACCACTTCACGTCCTC |
|  | AtHKT1;1 Rv | GGAAGATAAGTTGGGTGTTGGA |
|  | AtHKT1;1 P | CATCACCGTCTCTGGCATGTCTACCC |
|  |  |  |
