# Supplementary material for: Assessment of natural variation in the first pore domain of the tomato HKT1;2 transporter and characterization of mutated versions of SlHKT1;2 expressed in Xenopus laevis oocytes and via complementation of the salt sensitive athkt1;1 mutant
Source: Front Plant Sci. 2014 Nov 4;5:600. doi: 10.3389/fpls.2014.00600 (PMC4219482; doi:10.3389/fpls.2014.00600)
Supplement: Supplementary file 3 [file Table3.DOCX]

**Supplementary File Table 3:** Primers used to mutate *SlHKT1;2* and *AtHKT1;1*

| **Primer name** | **Primer sequence (5’ to 3’)** |
| --- | --- |
| SlHKT1;2-S70G Fw | CACAGTTTCTGGTATGTCCAC |
| SlHKT1;2-S70G Rv | GTGGACATACTAGAAACTGTG |
| AtHKT1;1-S68G Fw | CAAGAACCACTTCACGTCCTC |
| AtHKT1;1-S68G Rv | GGAAGATAAGTTGGGTGTTGGA |
