# Supplementary material for: Assessment of natural variation in the first pore domain of the tomato HKT1;2 transporter and characterization of mutated versions of SlHKT1;2 expressed in Xenopus laevis oocytes and via complementation of the salt sensitive athkt1;1 mutant
Source: Front Plant Sci. 2014 Nov 4;5:600. doi: 10.3389/fpls.2014.00600 (PMC4219482; doi:10.3389/fpls.2014.00600)
Supplement: Supplementary file 4 [file Table4.DOCX]

**Supplementary File Table 4:** List of primers used to amplify *AtHKT1;1* promoter and *AtHKT1;1*, *AtHKT1;1-S68G*, *SlHKT1;2* and *SlHKT1;2-S70G* genes with the *attB* Gateway recombination sites.

| **Gene/**  **Promoter** | **Primer name** | **Primer sequence (5’ to 3’)** |
| --- | --- | --- |
| AtHKT1;1 promotor | AtHKT1;1 prom (attB1) | GGGGACAAGTTTGTACAAAAAAGCAGGCTGCAAGTGATTGATACTCAGTTAAAAAGTTTCATCAAGC |
|  | AtHKT1;1 prom (attB5r) | GGGGACAACTTTTGTATACAAAGTTGTTTTAGTTCTCGAGTCGGTTTAAGCATTAA |
|  | AtHKT1;1 prom (attB2) | GGGGACCACTTTGTACAAGAAAGCTGGGTATTTAGTTCTCGAGTCGGTTTAAGCATTAA |
| AtHKT1;1 gene | AtHKT1;1 (attB5) | GGGGACAACTTTGTATACAAAAGTTGATGGACAGAGTGGTGGCA |
|  | AtHKT1;1 (attB2) | GGGGACCACTTTGTACAAGAAAGCTGGGTATTAGGAAGACGAGGGGTA |
| SlHKT1;2 gene | SlHKT1;2 (attB5) | GGGGACAACTTTGTATACAAAAGTTGATGAAGTCATCACTTTCA |
|  | SlHKT1;2 (attB2) | GGGGACCACTTTGTACAAGAAAGCTGGGTATTATAATACTTTCCAAGCC |
