# Supplementary figures and images for: Assessment of natural variation in the first pore domain of the tomato HKT1;2 transporter and characterization of mutated versions of SlHKT1;2 expressed in Xenopus laevis oocytes and via complementation of the salt sensitive athkt1;1 mutant
Source: Front Plant Sci. 2014 Nov 4;5:600. doi: 10.3389/fpls.2014.00600 (PMC4219482; doi:10.3389/fpls.2014.00600)

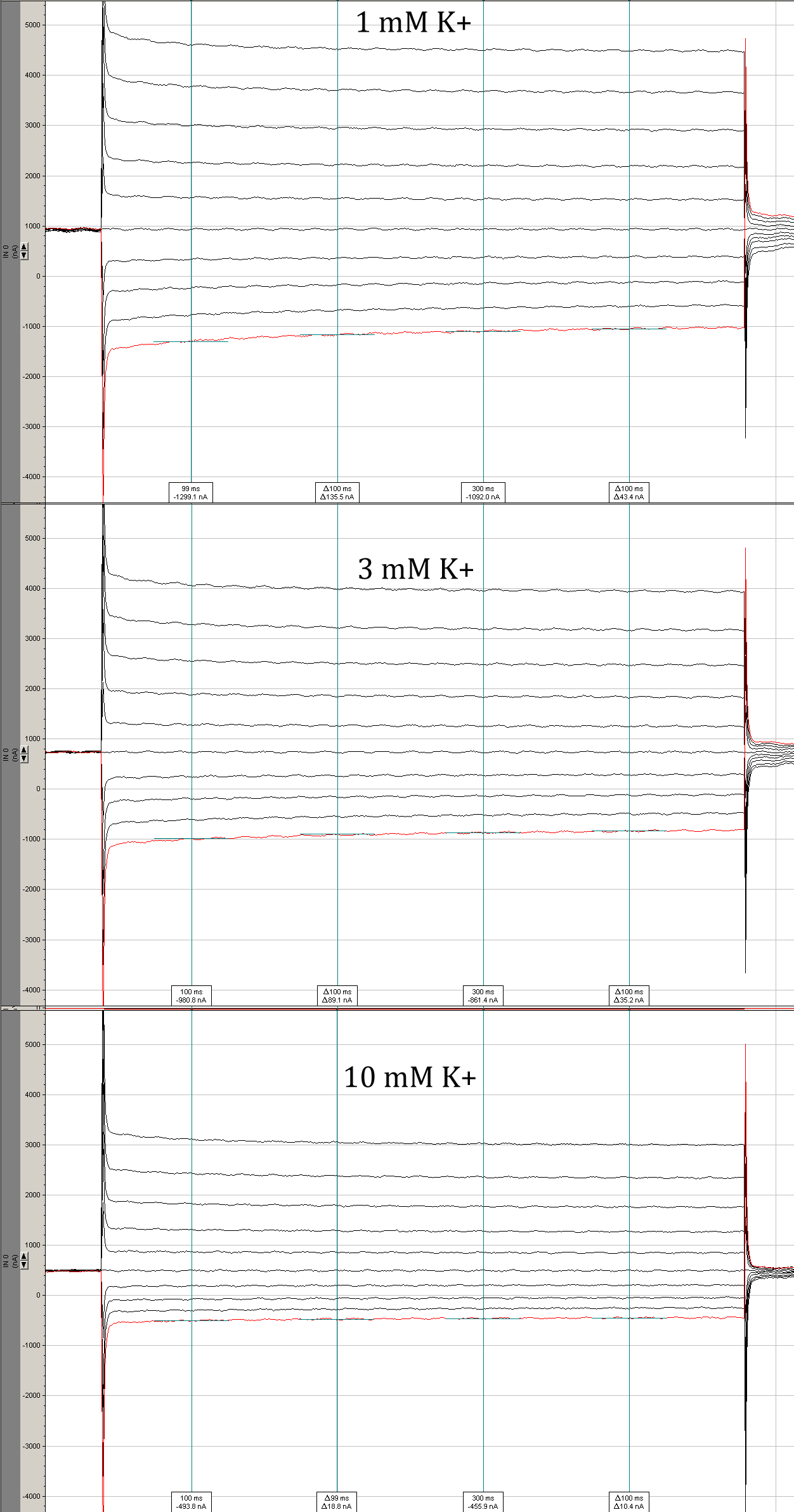

Supplement: Supplementary file 5 [file Image1.TIF]
